# Spatial organization of HPV-associated immune programs across tissue compartments in oropharyngeal cancer

**DOI:** 10.1371/journal.pone.0356717

**Published:** 2026-10-06

**Authors:** Naohiro Wakisaka, Makiko Moriyama-Kita, Satoru Kondo, Yosuke Nakanishi, Kazuhira Endo, Hisashi Sugimoto, Tomokazu Yoshizaki

**Affiliations:** 1 Department of Otorhinolaryngology, NHO Kanazawa Medical Center, Kanazawa, Japan; 2 Division of Otorhinolaryngology and Head and Neck Surgery, Graduate School of Medical Science, Kanazawa University, Kanazawa, Japan; University of Nebraska-Lincoln, UNITED STATES OF AMERICA

## Abstract

**Background:**

Human papillomavirus (HPV)-positive oropharyngeal cancer (OPC) is characterized by a distinct immune microenvironment; however, the biological significance of immune-related transcriptional programs may vary according to their spatial localization. We investigated the spatial organization of HPV-associated transcriptional programs across anatomically distinct tissue compartments in OPC.

**Methods:**

An oropharynx-restricted The Cancer Genome Atlas (TCGA)-oropharyngeal squamous cell carcinoma (OPSCC) cohort comprising 77 tumors (51 HPV-positive and 26 HPV-negative) was analyzed using single-sample gene set enrichment analysis (ssGSEA). Spatial transcriptomic profiling using GeoMx Digital Spatial Profiling was evaluated in lymphoid follicular regions (LFRs) and tumor tissue regions (TTRs) from six OPC patients (four HPV-positive and two HPV-negative), with the patient treated as the primary experimental unit. Tumor-free surrounding palatine tonsillar tissue (PTT) was analyzed in an independent microarray cohort comprising 23 HPV-positive and four HPV-negative cases. Eight prespecified immune- and metabolism-related transcriptional programs were evaluated, with additional exploratory cell-type deconvolution and overall survival analyses.

**Results:**

In TCGA-OPSCC, all five immune-related programs were higher in HPV-positive tumors, and Stem-like, Tpex, B-cell activity, and T-cell activity remained significantly associated with HPV-positive status after adjustment for clinicopathological covariates and FDR correction. In paired GeoMx analyses, all five immune-related programs were higher in LFRs than in TTRs in all six patients, whereas Estrogen Response Early and Estrogen Response Late were higher in TTRs in all six patients (BH-FDR = 0.0357 for each program). Bile Acid Metabolism showed no consistent compartment-specific difference. HPV-associated differences within individual GeoMx compartments and HPV-by-compartment interactions were not statistically significant after FDR correction. In PTT, immune-related and metabolic programs showed directional HPV-associated differences, but none remained significant after FDR correction. Higher scores for several immune-related programs were associated with improved overall survival in univariable TCGA-OPSCC analyses, but these associations were attenuated after accounting for HPV status.

**Conclusions:**

HPV-associated immune transcriptional programs identified in bulk OPC show consistent spatial organization across lymphoid follicular and tumor tissue compartments. The most robust spatial feature was within-patient compartmentalization between LFRs and TTRs, whereas evidence for HPV-associated differences within individual spatial compartments was limited. These findings emphasize the importance of anatomical context when interpreting transcriptional programs in OPC.

## Introduction

Human papillomavirus (HPV)-positive oropharyngeal cancer (OPC) is a distinct subtype of head and neck squamous cell carcinoma (HNSCC) characterized by favorable clinical outcomes and enhanced responsiveness to radiotherapy and systemic treatment compared with HPV-negative disease [1–3]. These clinical differences are closely associated with a unique tumor immune microenvironment, including increased infiltration of T cells, B cells, and tertiary lymphoid structures (TLS), which contribute to effective antitumor immunity and improved prognosis [4–7]. As immune-related transcriptional biomarkers are increasingly incorporated into precision oncology, understanding the biological significance of these immune programs has become essential for patient stratification and the development of immunotherapeutic strategies.

Recent transcriptomic studies have identified characteristic HPV-associated immune programs, including Stem-like, progenitor-exhausted (Tpex), cytotoxic T-cell, and B-cell signatures, primarily through bulk RNA sequencing and single-cell transcriptomic analyses [8–13]. However, these approaches provide limited information regarding the anatomical localization of these immune programs within tumor-associated tissues. Because bulk transcriptomic profiles represent averaged signals derived from heterogeneous tissue compartments, identical transcriptional signatures may reflect biologically distinct processes depending on where they are expressed. Consequently, interpretation of immune-related biomarkers based solely on bulk transcriptomic data may overlook spatial heterogeneity. Spatially resolved transcriptomic approaches therefore offer an important opportunity to determine not only which immune programs are present, but also where they are organized within the tumor ecosystem, thereby providing a more biologically informative framework for biomarker interpretation [6,14–16].

Despite substantial advances in the characterization of HPV-associated immune programs, their spatial organization across anatomically distinct tumor-associated tissues remains poorly understood. Previous transcriptomic and single-cell studies have primarily focused on bulk tumors or dissociated cellular populations, providing limited information regarding the anatomical compartments in which these transcriptional programs are represented [8–13]. This limitation is particularly relevant in OPC because tumor-associated tissues comprise multiple biological compartments with distinct cellular compositions and functional properties, including lymphoid follicular regions (LFRs), tumor tissue regions (TTRs), and tumor-free surrounding palatine tonsillar tissue (PTT). We therefore integrated bulk transcriptomic data from an oropharynx-restricted The Cancer Genome Atlas (TCGA)- oropharyngeal squamous cell carcinoma (OPSCC) cohort, GeoMx Digital Spatial Profiling (DSP) of LFRs and TTRs, and microarray analyses of tumor-free surrounding PTT. Through this multi-compartment approach, we sought to determine how HPV-associated transcriptional programs identified in bulk OPC are spatially organized across distinct anatomical compartments and whether their spatial distribution differs according to HPV status.

## Materials and methods

### Study design and analytical workflow

This study was designed to characterize the spatial organization of HPV-associated transcriptional programs across anatomically distinct tissue compartments in OPC. Three complementary transcriptomic datasets were integrated, comprising an oropharynx-restricted TCGA cohort, GeoMx DSP of LFRs and TTRs, and an independent microarray cohort of tumor-free surrounding PTTs, as summarized in Fig 1.

**Fig 1:**
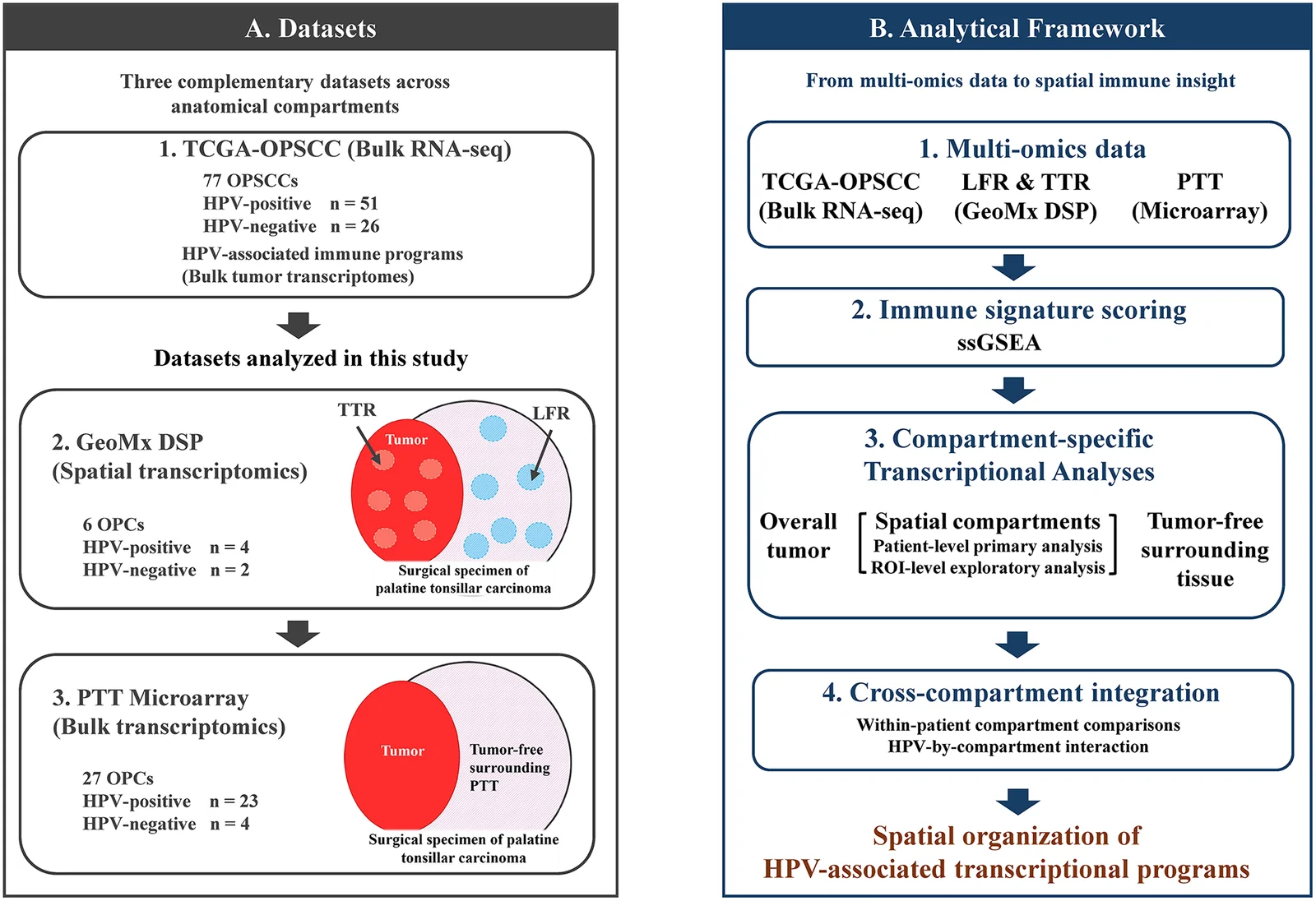
Study design and analytical framework. **(A)** Three complementary datasets were analyzed to characterize HPV-associated transcriptional programs across anatomical compartments in oropharyngeal cancer. The TCGA-OPSCC cohort comprised 77 tumors (51 HPV-positive and 26 HPV-negative) and was used to characterize HPV-associated transcriptional programs in bulk tumor transcriptomes. GeoMx Digital Spatial Profiling (DSP) was performed in six oropharyngeal cancers (four HPV-positive and two HPV-negative), with lymphoid follicular regions (LFRs) and tumor tissue regions (TTRs) analyzed as spatially distinct compartments. LFRs represented lymphoid follicular regions within palatine tonsillar tissue and included a B-cell follicular component and adjacent T-cell zone. The PTT microarray cohort comprised tumor-free surrounding palatine tonsillar tissue from 27 cases (23 HPV-positive and four HPV-negative). **(B)** Analytical framework. Transcriptional programs were quantified using single-sample gene set enrichment analysis (ssGSEA). For GeoMx DSP, patient-level analyses were used as the primary inferential approach, whereas ROI-level analyses were considered exploratory. Spatial organization was further assessed using within-patient LFR-versus-TTR comparisons and HPV-by-compartment interaction analyses. Findings across TCGA-OPSCC, GeoMx DSP, and PTT were integrated to characterize the spatial organization of HPV-associated transcriptional programs.

First, RNA-sequencing data from the oropharynx-restricted TCGA-OPSCC cohort were analyzed to define transcriptional programs associated with HPV status at the bulk-tumor level. Second, GeoMx DSP was used to examine the spatial organization of these programs across LFRs and TTRs. Because multiple regions of interest (ROIs) were obtained from each patient, the patient was treated as the primary experimental unit for statistical inference. Spatial organization within the GeoMx cohort was further evaluated by paired within-patient comparisons between LFRs and TTRs and by HPV-by-compartment interaction analyses. Third, microarray data from tumor-free surrounding PTT were analyzed as an exploratory assessment of the transcriptional context of surrounding tonsillar tissue.

Transcriptional programs were quantified using single-sample gene set enrichment analysis (ssGSEA). Across analyses, statistical inference was based on the appropriate independent experimental unit for each dataset, and multiple testing was controlled using the Benjamini–Hochberg false discovery rate (BH-FDR) procedure as described below. Additional exploratory analyses included reference-based cell-type deconvolution of GeoMx LFRs and overall survival analyses in the TCGA-OPSCC cohort.

The study was conducted under institutional ethical approval from the Ethics Committee of Kanazawa University (Approval No. 2016–033) as part of a broader research protocol entitled “Human genome and gene analysis of head and neck malignant tumors,” with an approved study period from March 29, 2016, to March 31, 2031. Written informed consent was obtained from all enrolled patients.

### TCGA-OPSCC dataset and HPV annotation

RNA-sequencing data and corresponding clinical information were obtained from TCGA-Head and Neck Squamous Cell Carcinoma (HNSC) dataset [17]. To provide an anatomically matched bulk-tumor reference for the spatial analyses, the present study was restricted to tumors arising from oropharyngeal subsites. The resulting TCGA-OPSCC cohort comprised 77 tumors, including 40 tonsillar tumors, 25 base-of-tongue tumors, 11 tumors classified as oropharynx not otherwise specified, and one posterior pharyngeal wall tumor. Gene-expression data were converted to gene-symbol-based expression profiles for downstream analyses.

HPV status was assigned using the RNA-seq-based HPV annotation reported by Nulton et al. [18]. Tumors annotated as HPV16-, HPV33-, HPV35-, or HPV56-positive were grouped as HPV-positive for the present analysis. The final TCGA-OPSCC cohort comprised 51 HPV-positive and 26 HPV-negative tumors.

Available clinicopathological characteristics were summarized according to HPV status. Age was compared using the Wilcoxon rank-sum test, whereas sex/gender, smoking status, clinical stage, and anatomical subsite were compared using Fisher’s exact test. These comparisons were considered descriptive cohort characterization and were not included in the Benjamini–Hochberg false discovery rate (BH-FDR) testing families used for transcriptional-program analyses.

Primary comparisons of transcriptional program scores between HPV-positive and HPV-negative tumors were performed in all 77 cases. To assess whether HPV-associated differences were retained after accounting for relevant clinicopathological factors, multivariable analyses were additionally performed using complete cases with available covariate data (n = 74; 50 HPV-positive and 24 HPV-negative). These models included age, sex/gender, smoking status, clinical stage, and anatomical subsite as covariates.

### Gene signature collection and ssGSEA analysis

Eight prespecified transcriptional programs were evaluated across the datasets. Five immune-related programs were included: a Stem-like T-cell-associated program, a precursor-exhausted T-cell-associated (Tpex) program, T-cell activity, B-cell activity, and a terminally exhausted T-cell-associated (Tex-like) program. The Stem-like, Tpex, T-cell activity, and B-cell activity signatures were constructed from established marker-gene sets, whereas the Tex-like signature was adopted from Shi et al. [19]. Three additional programs—Hallmark Estrogen Response Early, Hallmark Estrogen Response Late, and Hallmark Bile Acid Metabolism—were obtained from the Hallmark collection of the Molecular Signatures Database (MSigDB) [20]. The complete gene sets and the genes represented in the analyzed expression data are provided in S1 Table.

Single-sample gene set enrichment analysis (ssGSEA) was performed using the GSVA package in R [21]. Gene sets were matched to the available expression features within each dataset before ssGSEA calculation. Enrichment scores were calculated separately for each TCGA-OPSCC tumor, each GeoMx region of interest (ROI), and each PTT sample. For GeoMx DSP, ROI-level ssGSEA scores were subsequently averaged within each patient and anatomical compartment to generate patient-level scores for primary statistical analyses. Because ssGSEA scores are dependent on the underlying expression dataset and platform, absolute enrichment scores were not directly compared across TCGA RNA sequencing, GeoMx DSP, and PTT microarray datasets; instead, each dataset was analyzed separately, and the resulting patterns were integrated qualitatively.

### Complementary marker-gene analysis

As a complementary analysis of the Stem-like T-cell-associated transcriptional program identified by ssGSEA, eight prespecified marker genes (TCF7, IL7R, CCR7, LEF1, BACH2, SELL, SLAMF6, and CD28) were evaluated in the TCGA-OPSCC cohort. Expression levels were compared between HPV-positive and HPV-negative tumors, and P values were adjusted across the eight genes using the BH-FDR procedure. This marker-gene analysis was considered complementary to, rather than an independent validation of, the predefined multigene Stem-like T-cell-associated transcriptional program.

### GeoMx DSP dataset and compartment definition

Spatial transcriptomic analysis was performed using the NanoString GeoMx DSP on formalin-fixed, paraffin-embedded OPC specimens from six patients, comprising four HPV-positive and two HPV-negative cases. For each patient, six LFR ROIs and six TTR ROIs were analyzed, yielding 12 ROIs per patient and 72 ROIs in total.

ROI selection was performed independently by two investigators (M.M.-K. and N.W.) who were blinded to HPV and nodal status. PanCK, CD45, and SMA morphology markers were used to assist identification of epithelial/tumor, immune, and stromal compartments.

LFRs were defined histologically within palatine tonsillar tissue as lymphoid follicular regions containing a B-cell follicular component together with the adjacent T-cell zone. Thus, each LFR ROI represented a mixed lymphoid region rather than a purified B-cell or T-cell population. LFRs were selected within the lymphoid architecture of the palatine tonsil and were not classified as tumor-induced tertiary lymphoid structures (TLSs). TTRs were defined as regions containing carcinoma tissue and were selected separately from LFRs.

### GeoMx DSP data processing and statistical unit

GeoMx gene-expression data were processed using the GeoMx DSP Data Analysis Suite with Q3 normalization, as described previously [16]. All 72 selected ROIs were retained for the present analyses, with no patient samples or ROIs excluded after data processing. ssGSEA scores for the eight prespecified transcriptional programs were calculated separately for each ROI.

Because six ROIs from each compartment were obtained from each patient, ROI-level observations within a patient were not treated as independent biological replicates. The patient was therefore defined as the primary experimental unit for statistical inference. For each patient, the six ROI-level ssGSEA scores within each compartment were averaged to generate one patient-level LFR score and one patient-level TTR score for each transcriptional program. HPV-associated comparisons within each compartment were based primarily on these patient-level scores. ROI-level analyses were considered exploratory and were used to characterize within-patient and within-compartment variability rather than for primary statistical inference.

### Paired compartment and HPV-by-compartment interaction analyses

To directly assess transcriptional differences between LFRs and TTRs while accounting for the paired study design, patient-level compartment scores were compared within patients. For each transcriptional program, the within-patient difference was defined as the mean LFR score minus the mean TTR score. These paired differences were evaluated across the six patients using a two-sided exact Wilcoxon signed-rank test. P values were adjusted across the eight prespecified transcriptional programs using the BH-FDR procedure.

To determine whether the magnitude of the LFR–TTR difference varied according to HPV status, HPV-by-compartment interactions were evaluated using an exact patient-label permutation test. For each transcriptional program, the patient-level compartment difference was defined as Δ = mean LFR score − mean TTR score, and the interaction statistic was calculated as the difference in mean Δ between HPV-positive and HPV-negative patients. Given the four HPV-positive and two HPV-negative patients, all 15 possible assignments of four of the six patients to the HPV-positive group were enumerated. Two-sided exact permutation P values were calculated as the proportion of the 15 permuted statistics whose absolute values were greater than or equal to the absolute value of the observed statistic. P values were adjusted across the eight prespecified transcriptional programs using the BH-FDR procedure.

### PTT microarray dataset

Microarray gene-expression data from tumor-free surrounding PTT were obtained from the publicly available GSE228432 dataset. The dataset comprised 27 OPC patients, including 23 HPV-positive and four HPV-negative cases. PTT samples represented peritumoral PTT from which the tumor-containing region had been excluded by macrodissection of FFPE tissue sections before RNA extraction, as described previously [22]. The designation “tumor-free” therefore refers operationally to exclusion of the identified tumor-containing region and does not imply systematic microscopic exclusion of occult epithelial abnormalities, inflammatory infiltrates, or lymphoid structures.

The PTT dataset was analyzed separately as an exploratory assessment of the transcriptional context of tumor-free surrounding tonsillar tissue. ssGSEA scores for the same eight prespecified transcriptional programs used in the TCGA-OPSCC and GeoMx analyses were calculated for each PTT sample. Because of the marked imbalance in sample size between the HPV-positive and HPV-negative groups, particularly the small number of HPV-negative cases, findings from this dataset were considered exploratory and were interpreted on the basis of effect direction, effect size, and multiple-testing-adjusted P values.

### SpatialDecon analysis

To explore whether HPV-associated differences in the Stem-like T-cell-associated transcriptional program within LFRs could be related to differences in broad immune-cell composition, reference-based cell-type deconvolution was performed on the 36 LFR ROIs using SpatialDecon (version 1.18.0) [23] with the ImmuneTumor_safeTME reference matrix. Cell-type abundance estimates were obtained for each ROI and summarized into broad immune-cell categories, including B cells, CD4 T cells, CD8 T cells, and total T cells. For patient-level analyses, estimated abundances were averaged across the six LFR ROIs from each patient.

Patient-level cell-type estimates were compared between HPV-positive and HPV-negative patients. Associations between the patient-level Stem-like program score and the estimated abundance of each broad immune-cell category were additionally assessed using Spearman rank correlation. As exploratory sensitivity analyses, linear regression models were used to evaluate the association between HPV status and the Stem-like program score after adjustment separately for estimated CD8 T-cell abundance and total T-cell abundance. Given the small number of patients (n = 6), the deconvolution, correlation, and composition-adjusted analyses were considered exploratory and were not interpreted as establishing a cell-type-specific origin or composition-independent effect of the Stem-like transcriptional program.

### Overall survival analysis in TCGA-OPSCC

Overall survival was explored in the TCGA-OPSCC cohort using Cox proportional hazards regression. Overall survival time was defined from the available TCGA clinical follow-up data, with death from any cause treated as the event and patients alive at last follow-up censored. For each transcriptional program, ssGSEA scores were standardized to a mean of 0 and standard deviation of 1, and hazard ratios (HRs) were estimated per 1-standard-deviation increase in score.

Univariable Cox models were fitted separately for each of the eight prespecified transcriptional programs. To assess whether associations between transcriptional program scores and overall survival were retained after accounting for HPV status, additional Cox models including HPV status as a covariate were fitted for each program. The proportional hazards assumption was assessed for the Cox models. Because evidence of non-proportional hazards was observed for HPV status, HPV-stratified Cox analyses were additionally performed as sensitivity analyses. Given the limited number of deaths and the exploratory nature of these analyses, no additional multivariable clinicopathological adjustment was performed. P values were adjusted across the eight transcriptional programs within each survival-analysis family using the BH-FDR procedure.

### Statistical analysis

All statistical tests were two-sided. Comparisons between independent HPV-positive and HPV-negative groups were performed using the Wilcoxon rank-sum test, with exact P values used where appropriate. Effect sizes for group comparisons were quantified using Cliff’s delta (δ), with bootstrap 95% confidence intervals. Paired comparisons between patient-level LFR and TTR scores were performed using two-sided exact Wilcoxon signed-rank tests, as described above.

For GeoMx analyses, the patient rather than the individual ROI was treated as the primary experimental unit. Patient-level compartment scores were calculated by averaging the six ROI-level scores within each compartment for each patient. ROI-level analyses were considered exploratory and were not treated as independent biological replicates for primary statistical inference. HPV-by-compartment interactions were evaluated using the exact patient-label permutation procedure described above.

For the TCGA-OPSCC cohort, multivariable linear regression was used to evaluate associations between HPV status and each transcriptional program after adjustment for age, sex/gender, smoking status, clinical stage, and anatomical subsite. Overall survival analyses were performed using Cox proportional hazards regression as described above.

Multiple testing was controlled using the BH-FDR procedure. FDR correction was performed separately within each prespecified family of eight transcriptional-program tests for a given dataset and analysis, rather than across different datasets or distinct analytical questions. BH-adjusted P values < 0.05 were considered statistically significant. Results with nominal P < 0.05 but BH-adjusted P ≥ 0.05 were described as nominal associations and were not considered statistically significant. Raw and BH-adjusted P values are reported where applicable.

Statistical analyses were performed in R. Given the small number of patients in the GeoMx cohort, HPV-associated comparisons and interaction analyses were interpreted cautiously, with consideration of effect sizes, uncertainty, consistency of direction across patients, and FDR-adjusted statistical evidence.

## Results

### HPV-positive tumors exhibit coordinated immune-program enrichment in the TCGA-OPSCC cohort

To characterize HPV-associated transcriptional programs in an anatomically matched bulk-tumor cohort, we analyzed 77 tumors from the oropharynx-restricted TCGA-OPSCC cohort, comprising 51 HPV-positive and 26 HPV-negative tumors. Clinicopathological characteristics of the TCGA-OPSCC cohort according to HPV status are summarized in S2 Table. Age, sex/gender, and clinical stage did not differ significantly between HPV-positive and HPV-negative tumors (P = 0.304, P = 0.432, and P = 1.000, respectively). Smoking status showed a nonsignificant difference between groups (P = 0.064). In contrast, anatomical subsite differed significantly according to HPV status (P = 0.005), with tonsillar tumors more frequently represented among HPV-positive cases. In unadjusted comparisons, all five prespecified immune-related programs showed higher ssGSEA scores in HPV-positive tumors (Fig 2A and S3A Table). The corresponding effect sizes were largest for B-cell activity (Cliff’s δ = 0.618), followed by T-cell activity (δ = 0.561), the Stem-like T-cell-associated program (δ = 0.534), Tpex (δ = 0.499), and the Tex-like program (δ = 0.407). All five immune-related programs remained statistically significant after BH-FDR correction. In contrast, Estrogen Response Early, Estrogen Response Late, and Bile Acid Metabolism showed no significant HPV-associated differences.

**Fig 2:**
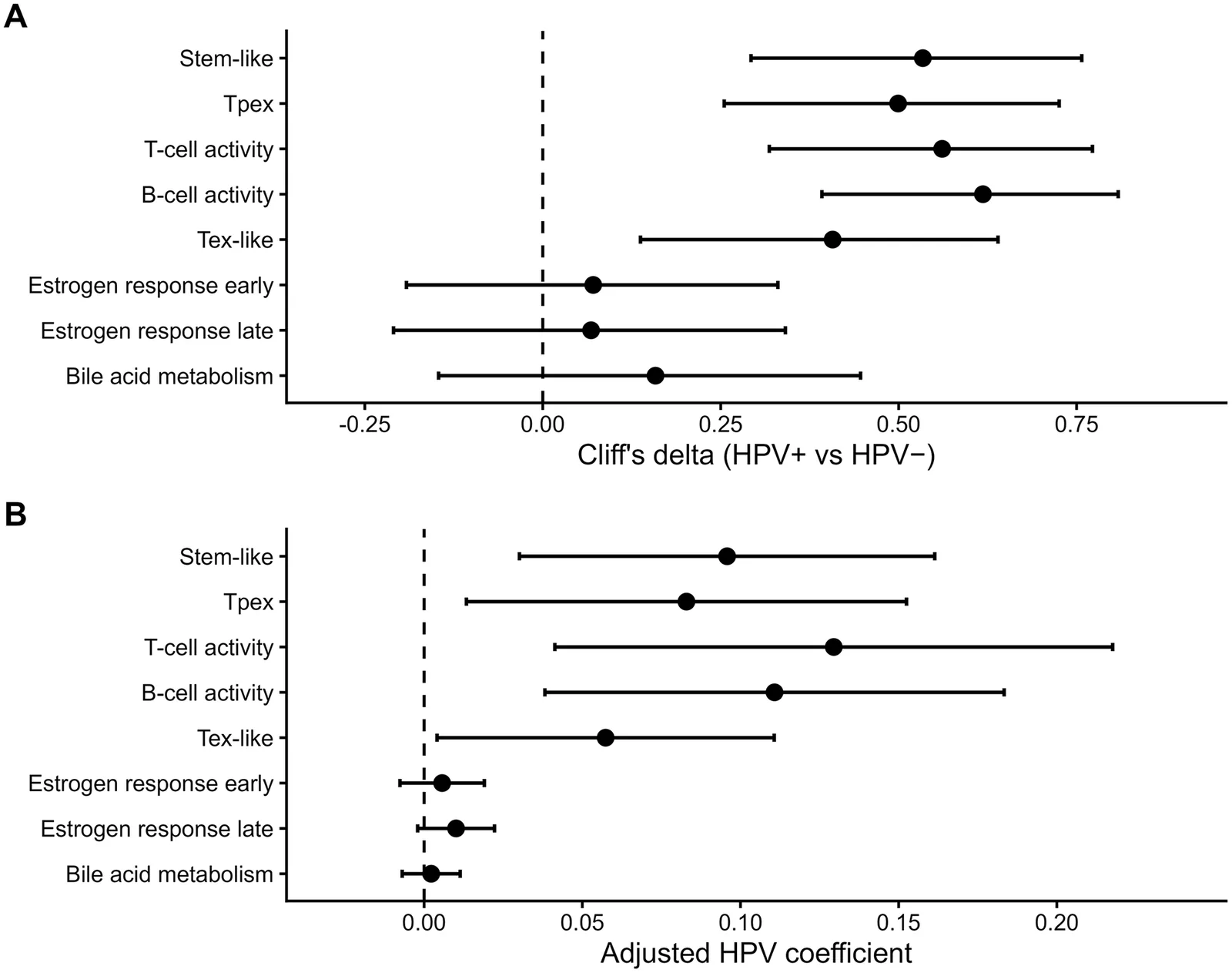
HPV-associated transcriptional programs in the TCGA-OPSCC cohort. **(A)** Unadjusted HPV-associated differences in ssGSEA scores in the oropharynx-restricted TCGA-OPSCC cohort (n = 77; HPV-positive, n = 51; HPV-negative, n = 26). Points indicate Cliff’s delta for HPV-positive versus HPV-negative tumors, and horizontal lines indicate bootstrap 95% confidence intervals. Positive values indicate higher scores in HPV-positive tumors. The five immune-related programs (Stem-like, Tpex, T-cell activity, B-cell activity, and Tex-like) showed positive HPV-associated effect sizes and remained significant after Benjamini–Hochberg false discovery rate (BH-FDR) correction, whereas estrogen-response and bile acid metabolism programs did not. **(B)** Multivariable-adjusted associations between HPV status and transcriptional program scores in complete cases (n = 74; HPV-positive, n = 50; HPV-negative, n = 24). Points indicate the regression coefficient for HPV-positive status and horizontal lines indicate 95% confidence intervals from linear models adjusted for age, sex, smoking status, clinical stage, and anatomical subsite. Positive coefficients indicate higher scores in HPV-positive tumors. Stem-like, Tpex, T-cell activity, and B-cell activity programs remained significantly associated with HPV-positive status after BH-FDR correction. The Tex-like program showed a nominal association but did not remain significant after FDR correction. Estrogen-response and bile acid metabolism programs were not significantly associated with HPV status after adjustment.

We next evaluated whether these associations were retained after adjustment for relevant clinicopathological factors. Multivariable linear regression analyses were performed in 74 complete cases (50 HPV-positive and 24 HPV-negative), with adjustment for age, sex/gender, smoking status, clinical stage, and anatomical subsite (Fig 2B and S3B Table). HPV-positive status remained significantly associated with higher scores for the Stem-like program (β = 0.0958, BH-FDR = 0.0130), Tpex (β = 0.0829, BH-FDR = 0.0404), B-cell activity (β = 0.1108, BH-FDR = 0.0130), and T-cell activity (β = 0.1295, BH-FDR = 0.0130). The Tex-like program showed a nominal positive association with HPV-positive status (β = 0.0574, nominal P = 0.0353) but did not remain significant after FDR correction (BH-FDR = 0.0565). The three Hallmark programs were not significantly associated with HPV status after multivariable adjustment.

Together, these findings identify a coordinated HPV-associated immune transcriptional phenotype in oropharyngeal tumors, with four immune-related programs remaining associated with HPV-positive status after adjustment for clinicopathological covariates.

### Complementary marker-gene analysis supports the HPV-associated Stem-like transcriptional program

As a complementary analysis of the Stem-like T-cell-associated transcriptional program, we examined eight prespecified marker genes (TCF7, IL7R, CCR7, LEF1, BACH2, SELL, SLAMF6, and CD28) in the TCGA-OPSCC cohort (S3C Table). Several of these genes showed higher expression in HPV-positive tumors. After multivariable adjustment for clinicopathological covariates and correction for multiple testing across the eight genes, CCR7, SELL, SLAMF6, and CD28 remained significantly associated with HPV-positive status. TCF7 showed a nominal association that did not remain statistically significant after FDR correction, whereas IL7R was not significantly associated with HPV status.

These findings were directionally consistent with the ssGSEA-based Stem-like transcriptional program and provide complementary gene-level context for the multigene signature. Given the overlap between these marker genes and the biological framework underlying the predefined signature, this analysis was not considered an independent validation of the Stem-like program.

### Immune and estrogen-response programs show consistent within-patient compartmentalization in GeoMx DSP

We next examined the spatial organization of the eight prespecified transcriptional programs across paired LFRs and TTRs using patient-level GeoMx DSP data. For each patient, ROI-level ssGSEA scores were averaged across the six ROIs within each compartment, yielding paired LFR and TTR scores for all six patients.

A strikingly consistent compartment-specific pattern was observed for the five immune-related programs (Fig 3A and S4A Table). Stem-like, Tpex, T-cell activity, B-cell activity, and Tex-like scores were higher in LFRs than in TTRs in all six patients. Each of these paired differences was statistically significant after correction for multiple testing (two-sided exact Wilcoxon signed-rank test, P = 0.03125; BH-FDR = 0.0357 for each program).

**Fig 3:**
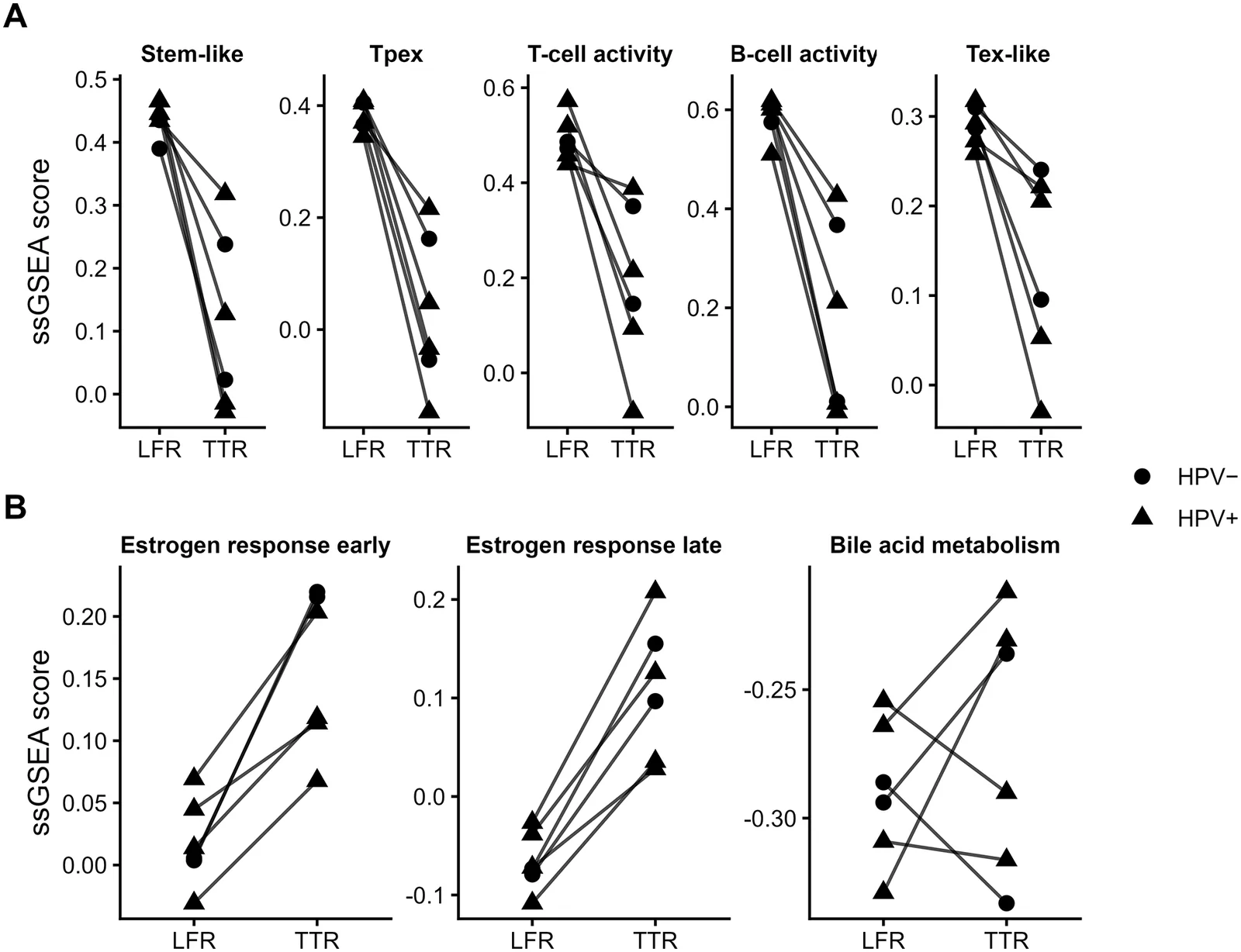
Within-patient compartmentalization of transcriptional programs in GeoMx DSP. **(A)** Patient-level ssGSEA scores for five immune-related transcriptional programs in lymphoid follicular regions (LFRs) and tumor tissue regions (TTRs) from six patients with oropharyngeal cancer. Each point represents the mean score across six ROIs from the corresponding compartment of an individual patient, and lines connect paired LFR and TTR measurements from the same patient. Circles indicate HPV-negative cases (n = 2) and triangles indicate HPV-positive cases (n = 4). Stem-like, Tpex, T-cell activity, B-cell activity, and Tex-like programs showed higher scores in LFRs than in TTRs in all six patients (two-sided exact Wilcoxon signed-rank test, BH-FDR = 0.036 for each program). **(B)** Patient-level ssGSEA scores for estrogen-response and bile acid metabolism programs in paired LFRs and TTRs. Estrogen response early and estrogen response late scores were higher in TTRs than in LFRs in all six patients (BH-FDR = 0.036 for each program). Bile acid metabolism showed no consistent compartment-specific direction (BH-FDR = 0.438). P values were adjusted across the eight prespecified transcriptional programs using the Benjamini–Hochberg procedure.

The opposite spatial pattern was observed for the two estrogen-response programs (Fig 3B and S4A Table). Estrogen Response Early and Estrogen Response Late scores were higher in TTRs than in LFRs in all six patients (P = 0.03125; BH-FDR = 0.0357 for each program). In contrast, Bile Acid Metabolism showed no consistent compartment-specific direction across patients and was not significantly different between LFRs and TTRs (P = 0.4375; BH-FDR = 0.4375).

These paired analyses demonstrate consistent spatial segregation of immune-related and estrogen-response transcriptional programs between lymphoid follicular and tumor tissue compartments within the same patients.

### HPV-associated differences within individual GeoMx compartments are suggestive but not definitive

We next examined whether transcriptional program scores within individual GeoMx compartments differed according to HPV status using patient-level scores. Comparisons were performed between four HPV-positive and two HPV-negative patients separately within LFRs and TTRs (Fig 4A and S4B Table). None of the eight prespecified transcriptional programs showed a statistically significant HPV-associated difference within either compartment after BH-FDR correction. Within LFRs, the Stem-like T-cell-associated program showed the largest positive HPV-associated effect among the immune-related programs (Cliff’s δ = 0.75), indicating higher patient-level scores in HPV-positive than in HPV-negative cases. However, this difference was not statistically significant (exact P = 0.267; BH-FDR = 1.00). Thus, although the direction of the Stem-like program was consistent with the HPV-associated immune phenotype observed in the TCGA-OPSCC cohort, the small GeoMx cohort did not provide definitive patient-level evidence for an HPV-associated difference within LFRs.

**Fig 4:**
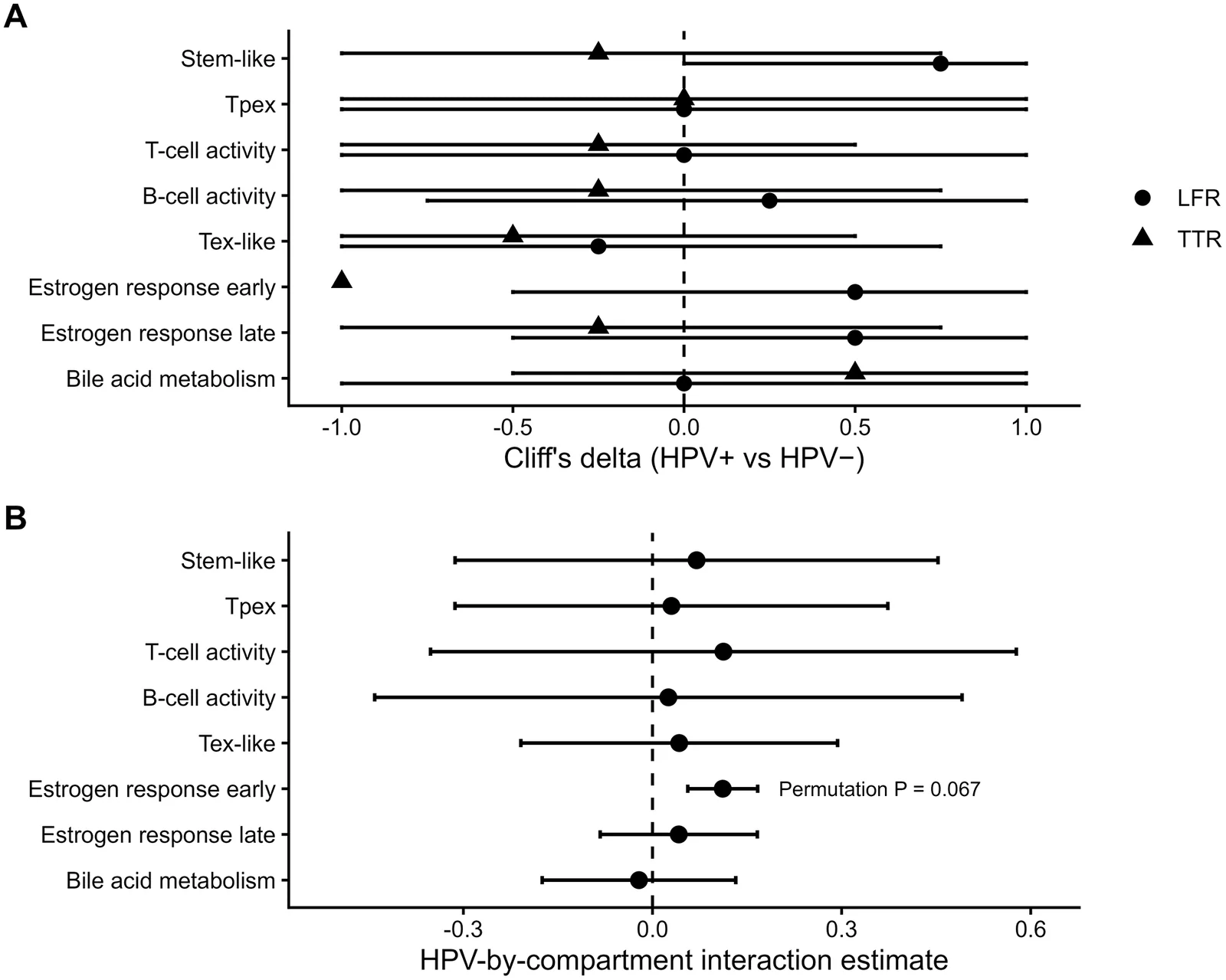
Patient-level HPV-associated effects and HPV-by-compartment interactions in GeoMx DSP. **(A)** Patient-level effect sizes for HPV-associated differences in ssGSEA scores within lymphoid follicular regions (LFRs) and tumor tissue regions (TTRs). Points indicate Cliff’s delta for HPV-positive versus HPV-negative patients (HPV-positive, n = 4; HPV-negative, n = 2), and horizontal lines indicate bootstrap 95% confidence intervals. Circles represent LFRs and triangles represent TTRs. Positive values indicate higher scores in HPV-positive patients. None of the eight prespecified transcriptional programs showed a statistically significant HPV-associated difference within either compartment after Benjamini–Hochberg false discovery rate (BH-FDR) correction. **(B)** HPV-by-compartment interaction analysis testing whether the within-patient LFR-versus-TTR difference varied according to HPV status. Points indicate interaction-effect estimates and horizontal lines indicate 95% confidence intervals from the corresponding linear models. Statistical inference was based on exact patient-label permutation tests across all possible allocations of four HPV-positive and two HPV-negative patients, with BH-FDR correction across the eight prespecified programs. No interaction remained statistically significant after FDR correction. Estrogen response early showed the strongest suggestive interaction (exact permutation P = 0.067; BH-FDR = 0.533).

We then tested whether the magnitude of the within-patient LFR-versus-TTR difference varied according to HPV status (Fig 4B and S4C Table). None of the eight HPV-by-compartment interactions remained statistically significant after BH-FDR correction. The strongest suggestive interaction was observed for Estrogen Response Early (interaction mean difference = 0.1114; exact permutation P = 0.0667; BH-FDR = 0.533), but this did not meet the prespecified threshold for statistical significance.

Collectively, these analyses indicate that HPV-associated differences within individual GeoMx compartments and HPV-dependent modification of LFR-versus-TTR differences were suggestive but not statistically definitive in this small patient cohort. In contrast, the within-patient compartmentalization observed across LFRs and TTRs was highly consistent across patients, irrespective of HPV status.

### Reference-based deconvolution does not identify a clear HPV-associated difference in broad LFR immune-cell composition

To explore whether the HPV-associated direction of the Stem-like T-cell-associated transcriptional program within LFRs could be related to differences in broad immune-cell composition, we performed reference-based cell-type deconvolution of the 36 LFR ROIs and summarized the resulting estimates at the patient level (S1A Fig and S5A Table). Estimated abundances of B cells, CD4 T cells, CD8 T cells, and total T cells did not differ significantly between HPV-positive and HPV-negative patients after BH-FDR correction.

We next examined associations between the patient-level Stem-like program score and deconvolution-derived immune-cell estimates. The Stem-like score was not positively associated with estimated CD8 T-cell abundance (Spearman ρ = −0.371, P = 0.468), whereas a positive association was observed with estimated total T-cell abundance (ρ = 0.829, P = 0.042) (S1B Fig and S5B Table). Associations with B-cell and CD4 T-cell estimates were not statistically significant.

As exploratory sensitivity analyses, we evaluated the HPV-associated direction of the Stem-like program after adjustment separately for estimated CD8 T-cell and total T-cell abundance (S5C Table). The HPV-associated coefficient remained positive after adjustment for CD8 T-cell abundance (β = 0.0387, 95% CI, −0.0175 to 0.0950; P = 0.116) and total T-cell abundance (β = 0.0351, 95% CI, −0.0267 to 0.0968; P = 0.169), but neither association was statistically significant.

Together, these exploratory analyses did not identify a clear HPV-associated difference in broad immune-cell composition within LFRs. Although broad cell-type composition alone may not fully account for the observed HPV-associated direction of the Stem-like transcriptional program, the small sample size precludes inference regarding composition-independent enrichment or the specific cellular origin of this program.

### Tumor-free surrounding tonsillar tissue shows directional but nonsignificant HPV-associated transcriptional patterns

We next examined the eight prespecified transcriptional programs in an independent microarray cohort of tumor-free surrounding PTT from 23 HPV-positive and four HPV-negative OPC cases. Given the small number of HPV-negative cases, these analyses were considered exploratory (Fig 5 and S6 Table).

**Fig 5:**
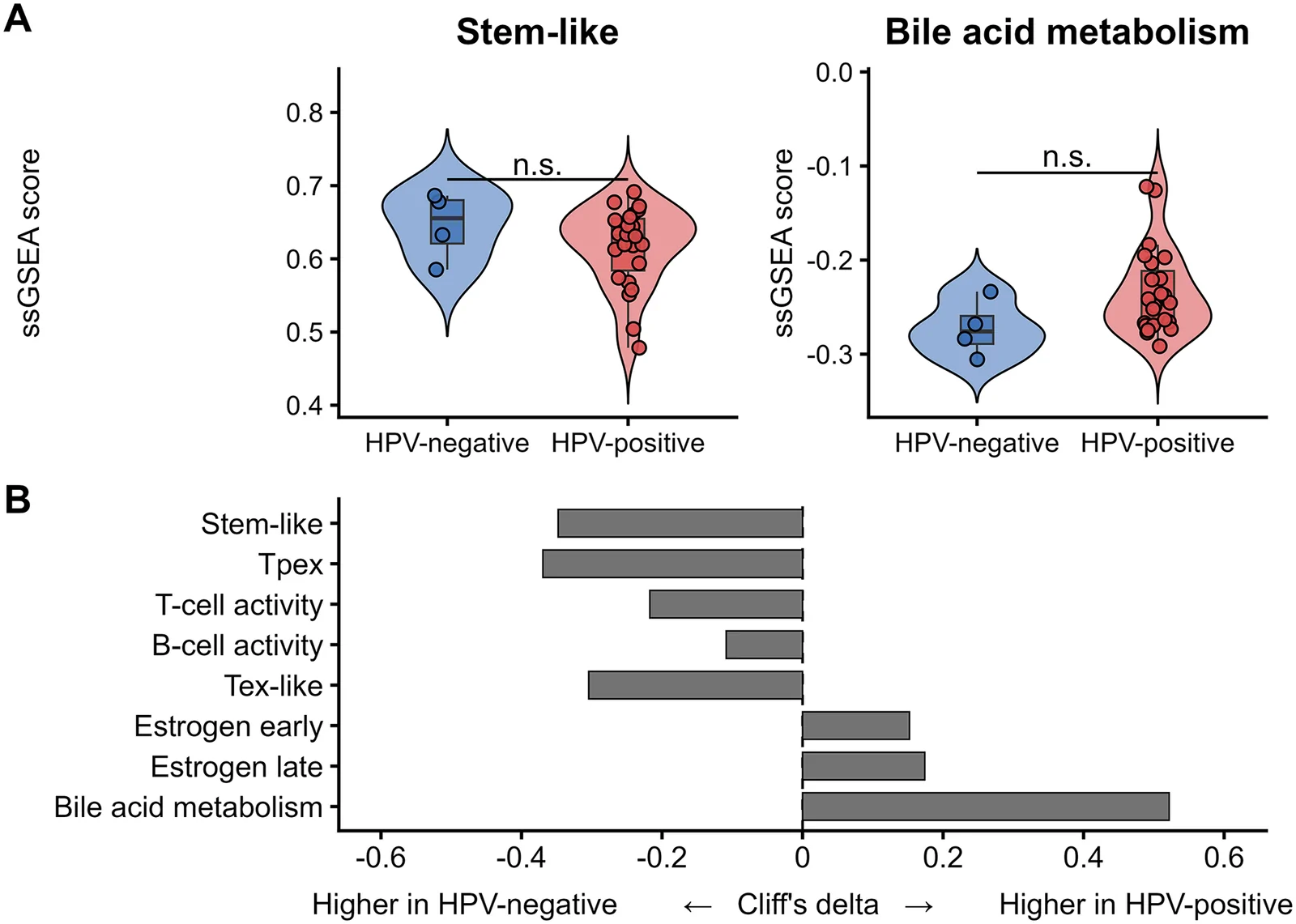
Transcriptional patterns in tumor-free surrounding palatine tonsillar tissue. **(A)** Representative ssGSEA scores for the Stem-like T-cell-associated program and bile acid metabolism program in tumor-free surrounding palatine tonsillar tissue (PTT) from HPV-positive (n = 23) and HPV-negative (n = 4) cases. Violin plots show the distribution of patient-level scores, with boxplots indicating the median and interquartile range and individual points representing patients. Group comparisons were performed using two-sided exact Wilcoxon rank-sum tests. Neither program showed a statistically significant difference between HPV groups. **(B)** Effect sizes for HPV-associated differences across the eight prespecified transcriptional programs in PTT. Bars indicate Cliff’s delta for HPV-positive versus HPV-negative cases. Positive values indicate higher scores in HPV-positive cases, whereas negative values indicate higher scores in HPV-negative cases. None of the eight transcriptional programs remained statistically significant after Benjamini–Hochberg false discovery rate correction. Given the small number of HPV-negative cases, these analyses were considered exploratory.

Representative comparisons of the Stem-like T-cell-associated and Bile Acid Metabolism programs are shown in Fig 5A. The Stem-like program showed a directional difference toward higher scores in HPV-negative PTT, whereas Bile Acid Metabolism showed a directional difference toward higher scores in HPV-positive PTT; neither difference was statistically significant.

Across all eight transcriptional programs, effect-size analysis showed a consistent directional pattern in which the five immune-related programs had negative Cliff’s delta values, indicating higher scores in HPV-negative PTT, whereas Estrogen Response Early, Estrogen Response Late, and Bile Acid Metabolism had positive effect sizes, indicating higher scores in HPV-positive PTT (Fig 5B and S6 Table). However, none of the eight programs remained statistically significant after BH-FDR correction.

Thus, the PTT dataset showed directional HPV-associated transcriptional differences in tumor-free surrounding tonsillar tissue, but these exploratory findings did not provide statistically significant evidence for HPV-associated differences after correction for multiple testing.

### Associations between immune transcriptional programs and overall survival are attenuated after accounting for HPV status

We additionally explored associations between the eight prespecified transcriptional programs and overall survival in the TCGA-OPSCC cohort (n = 77; 21 deaths) (S2A Fig and S7A Table). In univariable Cox analyses, higher scores for the Stem-like (HR = 0.501), Tpex (HR = 0.491), B-cell activity (HR = 0.490), and T-cell activity (HR = 0.571) programs were associated with improved overall survival after BH-FDR correction (BH-FDR = 0.0338, 0.0338, 0.0338, and 0.0350, respectively). The Tex-like program also showed a nominal association with improved overall survival (HR = 0.577, nominal P = 0.0383), but this association did not remain statistically significant after FDR correction (BH-FDR = 0.0614).

When HPV status was included as a covariate, the associations between transcriptional program scores and overall survival were attenuated, and none of the eight programs remained statistically significant after BH-FDR correction (S2B Fig and S7B Table). Because Schoenfeld residual-based testing indicated evidence of non-proportional hazards for HPV status, HPV-stratified Cox models were additionally evaluated as a sensitivity analysis. None of the eight transcriptional programs was significantly associated with overall survival after BH-FDR correction in these stratified models (S2C Fig and S7C Table).

Thus, although several immune-related transcriptional programs were associated with improved overall survival in univariable analyses, these exploratory analyses did not provide evidence that the evaluated programs were associated with overall survival beyond the prognostic information captured by HPV status.

### Integrated analysis reveals compartment-specific spatial organization of transcriptional programs

To integrate the findings from the TCGA-OPSCC, GeoMx DSP, and PTT datasets, we summarized the observed transcriptional patterns across bulk tumor, lymphoid follicular, tumor tissue, and tumor-free surrounding tonsillar compartments (Fig 6). In the TCGA-OPSCC cohort, HPV-positive tumors showed coordinated enrichment of multiple immune-related transcriptional programs, establishing a bulk-tumor reference for HPV-associated immune transcriptional features.

**Fig 6:**
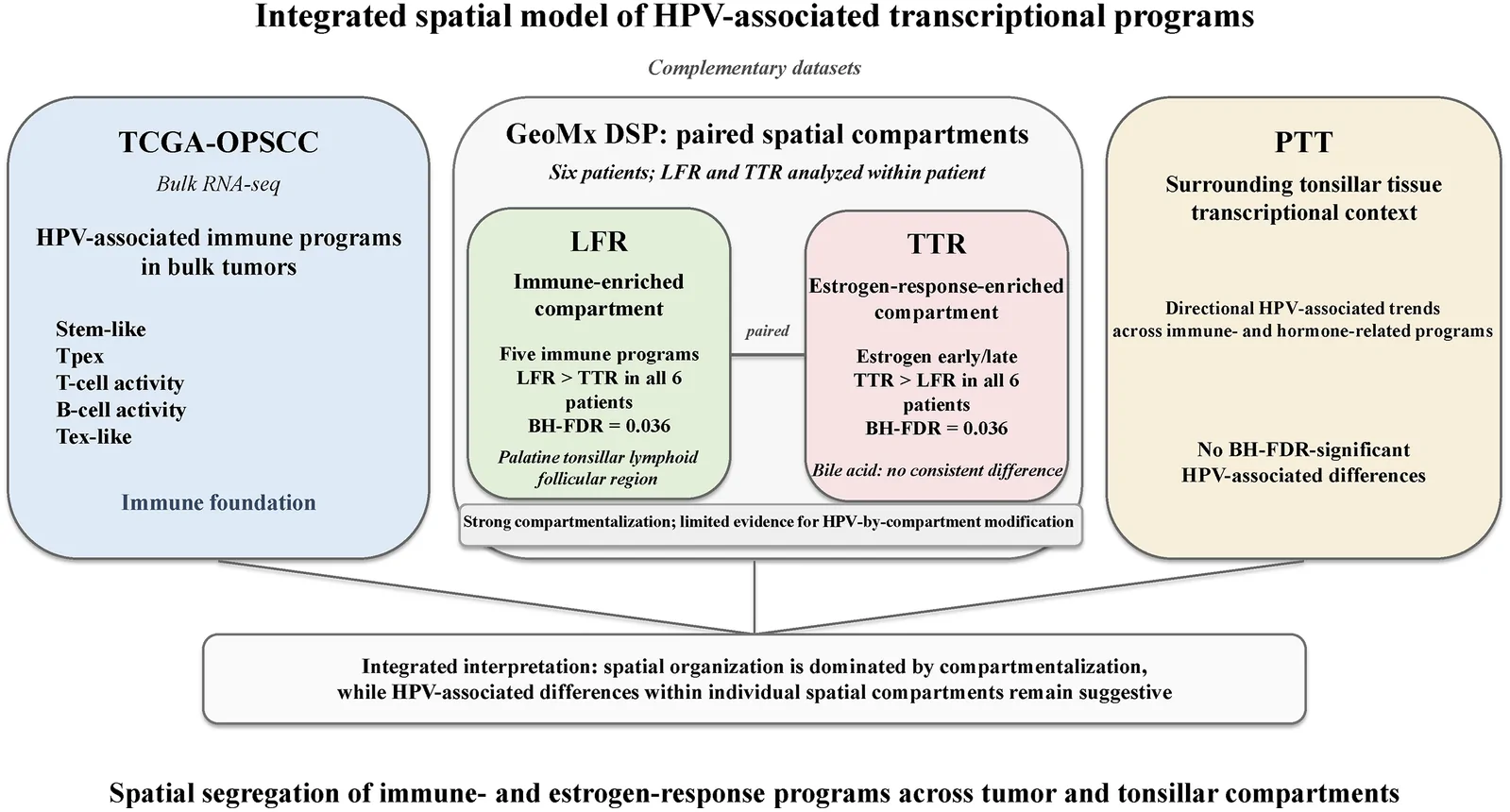
Integrated spatial model of HPV-associated transcriptional programs in oropharyngeal cancer. The integrated model summarizes complementary findings from TCGA-OPSCC bulk RNA sequencing, GeoMx DSP, and tumor-free surrounding palatine tonsillar tissue (PTT). In TCGA-OPSCC, HPV-positive tumors showed higher scores for multiple immune-related transcriptional programs, providing a bulk-tumor transcriptional foundation for subsequent spatial analyses. In GeoMx DSP, paired patient-level comparisons demonstrated strong compartmentalization: five immune-related programs (Stem-like, Tpex, T-cell activity, B-cell activity, and Tex-like) were consistently higher in lymphoid follicular regions (LFRs) than in tumor tissue regions (TTRs) in all six patients (BH-FDR = 0.036 for each), whereas estrogen response early and late were consistently higher in TTRs than in LFRs (BH-FDR = 0.036 for each). Bile acid metabolism showed no consistent compartment-specific difference. HPV-associated differences within individual GeoMx compartments and HPV-by-compartment interactions were not statistically significant after FDR correction, indicating limited evidence that HPV status modified the observed compartmentalization. In PTT, directional HPV-associated differences were observed across immune- and hormone-related programs, but none remained statistically significant after FDR correction. Together, these findings support spatial segregation of immune- and estrogen-response transcriptional programs across tumor and tonsillar compartments, while HPV-associated differences within individual spatial compartments remain suggestive.

Spatial analysis using GeoMx DSP demonstrated a consistent compartment-specific organization of these programs within individual patients. All five immune-related programs were higher in LFRs than in TTRs in each of the six patients, whereas Estrogen Response Early and Estrogen Response Late were consistently higher in TTRs. Bile Acid Metabolism showed no consistent compartment-specific difference. In contrast to these strong paired compartmental differences, HPV-associated differences within individual LFR or TTR compartments and HPV-by-compartment interactions were not statistically significant after FDR correction.

In tumor-free surrounding PTT, immune-related programs showed directional differences toward higher scores in HPV-negative cases, whereas estrogen-response and bile acid metabolism programs showed directional differences toward higher scores in HPV-positive cases; however, none remained statistically significant after FDR correction.

Together, these complementary datasets support a model in which immune-related and estrogen-response transcriptional programs are spatially segregated across lymphoid follicular and tumor tissue compartments in OPC. The most consistent spatial feature was compartmentalization between LFRs and TTRs, whereas evidence for HPV-associated differences within individual spatial compartments was limited in the small GeoMx cohort and findings in surrounding PTT remained exploratory.

## Discussion

The principal finding of the present study is that HPV-associated immune transcriptional programs identified in bulk OPC are spatially organized across anatomically distinct tissue compartments. In the oropharynx-restricted TCGA-OPSCC cohort, HPV-positive tumors showed coordinated enrichment of multiple immune-related programs, including Stem-like, Tpex, T-cell activity, and B-cell activity programs. Spatial analysis using GeoMx DSP further demonstrated that the most consistent feature was not an HPV-associated difference within an individual compartment, but rather a marked within-patient segregation between LFRs and TTRs: all five immune-related programs were higher in LFRs, whereas Estrogen Response Early and Estrogen Response Late were higher in TTRs. In contrast, HPV-associated differences within individual GeoMx compartments and HPV-by-compartment interactions were not statistically significant after FDR correction. These findings emphasize that transcriptional programs identified in bulk tissue should be interpreted in relation to their anatomical localization and support a compartment-aware framework for interpreting spatially resolved immune biomarkers in OPC [24–28].

Among the compartment-specific findings, the consistent enrichment of the Stem-like T-cell-associated transcriptional program in LFRs relative to TTRs was a prominent spatial feature. Previous studies have identified stem-like or progenitor-exhausted T-cell populations characterized by TCF7 and related markers that can sustain antitumor immune responses and generate differentiated effector populations during chronic antigen stimulation or immune checkpoint blockade [10–12,29]. Likewise, TLSs and other organized lymphoid aggregates can provide specialized microenvironments supporting antigen presentation and coordinated T- and B-cell interactions, and have been associated with favorable clinical outcomes and responses to immunotherapy in several cancer types [4,14,15,26,30]. In the present study, however, the Stem-like score represents a multigene transcriptional program rather than direct quantification of a defined stem-like CD8 T-cell population. Moreover, the LFRs analyzed here were lymphoid follicular regions within the lymphoid architecture of the palatine tonsil, containing a B-cell follicular component and adjacent T-cell zone, and were not classified as tumor-induced TLSs. Thus, the higher Stem-like program scores observed in LFRs should be interpreted as enrichment of a stem-like T-cell-associated transcriptional program within a lymphoid-rich anatomical compartment, rather than as direct evidence of increased numbers of stem-like CD8 T cells or the presence of tumor-induced TLSs.

The exploratory deconvolution analysis provides additional context for interpreting the Stem-like transcriptional program. Broad immune-cell composition within LFRs, including estimated B-cell, CD4 T-cell, CD8 T-cell, and total T-cell abundance, did not show significant HPV-associated differences after FDR correction. Moreover, the Stem-like program score was not positively associated with estimated CD8 T-cell abundance, although it was positively associated with estimated total T-cell abundance. In sensitivity analyses adjusting separately for CD8 T-cell or total T-cell abundance, the HPV-associated coefficient for the Stem-like program remained positive but was not statistically significant. These findings suggest that broad differences in estimated immune-cell abundance alone may not fully account for the observed transcriptional pattern. However, given the small number of patients and the reference-based nature of the deconvolution analysis, these results do not establish composition-independent enrichment or identify the specific cellular source of the Stem-like program. Higher-resolution approaches that directly resolve cellular identity and state will be required to determine which lymphoid populations contribute to this transcriptional signal.

The paired comparison between LFRs and TTRs further highlights the importance of anatomical compartment in interpreting transcriptional programs in OPC. All five immune-related programs were consistently higher in LFRs than in TTRs within the same patients, whereas Estrogen Response Early and Estrogen Response Late showed the opposite pattern and were consistently higher in TTRs. Bile Acid Metabolism, in contrast, showed no consistent compartment-specific difference. These findings extend previous observations that the head and neck tumor microenvironment contains spatially distinct immune niches and microenvironmental architectures [6,24,27,28]. Importantly, the higher estrogen-response scores in TTRs should be interpreted as compartment-specific transcriptional enrichment rather than evidence of a specific estrogen-dependent mechanism, because the present analysis does not resolve the cellular source or functional activity underlying these Hallmark signatures. Taken together, the paired findings support spatial segregation of immune-related and estrogen-response transcriptional programs between lymphoid-rich and tumor-containing compartments, emphasizing that the biological interpretation of transcriptomic signatures depends on the anatomical compartment in which they are measured [24,25,28].

Tumor-free surrounding PTT provided a complementary view of transcriptional programs outside the tumor-containing compartment. The five immune-related programs showed directional differences toward higher scores in HPV-negative PTT, whereas Estrogen Response Early, Estrogen Response Late, and Bile Acid Metabolism showed directional differences toward higher scores in HPV-positive PTT. However, none of these differences remained statistically significant after FDR correction. Accordingly, these findings do not provide evidence for a persistent HPV-associated immune field in surrounding tonsillar tissue, but suggest that anatomically adjacent tissues may provide complementary context for interpreting spatially heterogeneous transcriptional programs [24,28].

Taken together, these findings indicate that transcriptional programs identified in bulk tissue can exhibit distinct patterns across anatomical compartments. Anatomical context should therefore be considered when interpreting transcriptional signatures and designing future spatial biomarker and tissue-sampling studies [24,25,27,28].

This study has several limitations. First, the GeoMx DSP cohort comprised only six patients, including four HPV-positive and two HPV-negative cases, which substantially limited statistical power for detecting HPV-associated differences within individual compartments and HPV-by-compartment interactions. We therefore treated the patient as the primary experimental unit, while ROI-level analyses were considered exploratory because multiple ROIs obtained from the same patient are not statistically independent. Second, LFRs represented mixed lymphoid regions containing both B-cell follicular and adjacent T-cell-zone components, and the ssGSEA signatures used in this study reflect multigene transcriptional programs rather than direct measurements of specific immune-cell populations or functional states. Reference-based deconvolution provided additional information on broad immune-cell composition, but this analysis was exploratory and cannot resolve the precise cellular sources of the observed transcriptional programs. Third, the PTT cohort was markedly imbalanced with respect to HPV status, with 23 HPV-positive and only four HPV-negative cases, limiting inference from the observed directional differences. Fourth, the three datasets were generated using different transcriptomic platforms and sampled biologically distinct tissue contexts; accordingly, our integration focused on the direction and spatial organization of predefined transcriptional programs rather than direct comparison of absolute ssGSEA scores across datasets. In addition, HPV classification was not determined using an identical assay across the three datasets, and the TCGA-OPSCC classification was based on published RNA-seq-derived viral annotation that included multiple high-risk HPV types; this methodological heterogeneity should be considered when comparing HPV-associated patterns across datasets. Fifth, the survival analyses were exploratory, with only 21 deaths in the TCGA-OPSCC cohort. Associations between several immune programs and overall survival observed in univariable analyses were attenuated after accounting for HPV status, and the proportional-hazards assumption was not satisfied for HPV status. Thus, these analyses do not establish prognostic value independent of HPV status. Finally, no standardized cohort linking the evaluated transcriptional programs to response to immune checkpoint therapy was available in the datasets analyzed here. Larger independent spatial cohorts, ideally incorporating higher-resolution cellular measurements and treatment-response data, will therefore be required to validate the biological and clinical relevance of the observed compartment-specific transcriptional organization.

Future studies using higher-resolution spatial technologies will be important for identifying the cellular populations contributing to the Stem-like and other immune-related programs within LFRs. Larger independent spatial cohorts will also be required to determine the reproducibility and clinical relevance of the observed LFR–TTR compartmentalization.

## Conclusion

In summary, this study demonstrates that HPV-associated immune transcriptional programs identified in bulk OPC are spatially organized across distinct anatomical compartments. In the oropharynx-restricted TCGA-OPSCC cohort, HPV-positive tumors showed coordinated enrichment of multiple immune-related programs, whereas GeoMx DSP revealed consistent within-patient compartmentalization, with immune-related programs enriched in LFRs and estrogen-response programs enriched in TTRs. In contrast, HPV-associated differences within individual GeoMx compartments and HPV-by-compartment interactions were not statistically significant after FDR correction, and findings in tumor-free surrounding PTT remained exploratory. Together, these results identify anatomical compartment as an important context for interpreting transcriptional programs in OPC and support a compartment-aware framework for future spatial biomarker studies.

## Supporting information

S1 Fig Reference-based cell-type deconvolution of GeoMx lymphoid follicular regions. **(A)** Reference-based estimates of broad immune-cell composition within lymphoid follicular regions (LFRs) from six patients analyzed by GeoMx DSP. Cell-type abundances were estimated using SpatialDecon with the ImmuneTumor_safeTME reference and summarized at the patient level by averaging across six LFR ROIs per patient. Circles indicate HPV-negative patients (n = 2) and triangles indicate HPV-positive patients (n = 4); horizontal bars indicate group medians. Estimated proportions of B cells, CD4 T cells, CD8 T cells, and total T cells showed no statistically significant differences between HPV groups after Benjamini–Hochberg false discovery rate correction. **(B)** Association between patient-level Stem-like ssGSEA scores and estimated CD8 T-cell proportions within LFRs. No positive association was observed between the Stem-like program and estimated CD8 T-cell proportion (Spearman ρ = −0.371, P = 0.468). **(C)** Association between patient-level Stem-like ssGSEA scores and estimated total T-cell proportions within LFRs. A positive association was observed (Spearman ρ = 0.829, P = 0.042). Given the small patient-level sample size (n = 6), the correlation analyses were considered exploratory and were not interpreted as evidence of cellular independence or a specific cellular origin of the Stem-like transcriptional program.
(TIFF)

S2 Fig Exploratory overall survival analyses of transcriptional programs in the TCGA-OPSCC cohort. **(A)** Univariable Cox proportional hazards analyses of overall survival in the TCGA-OPSCC cohort (n = 77; 21 deaths). Hazard ratios (HRs) are shown per 1-standard-deviation increase in each ssGSEA score, with horizontal lines indicating 95% confidence intervals. Higher scores for the Stem-like, Tpex, B-cell activity, and T-cell activity programs were associated with improved overall survival after Benjamini–Hochberg false discovery rate (BH-FDR) correction. The Tex-like program showed a nominal association but did not remain significant after FDR correction. **(B)** Cox models including each standardized transcriptional program and HPV status. Associations between the transcriptional programs and overall survival were attenuated after accounting for HPV status, and none remained statistically significant after BH-FDR correction. HPV status showed evidence of non-proportional hazards based on Schoenfeld residuals. **(C)** HPV-stratified Cox sensitivity analyses, in which the baseline hazard was allowed to differ according to HPV status. None of the transcriptional programs was significantly associated with overall survival after BH-FDR correction. Points indicate HRs per 1-standard-deviation increase in ssGSEA score, and horizontal lines indicate 95% confidence intervals. The vertical dashed line indicates HR = 1. Overall, these exploratory analyses did not provide evidence that the evaluated transcriptional programs were associated with overall survival beyond the prognostic information captured by HPV status.
(TIFF)

S1 Table Gene sets used for single-sample gene set enrichment analysis. Eight prespecified transcriptional programs were evaluated, comprising five immune-related programs (Stem-like, Tpex, B-cell activity, T-cell activity, and Tex-like) and three Hallmark programs (estrogen response early, estrogen response late, and bile acid metabolism). The table lists the gene-set name, source, original number of genes, number of genes available for analysis in the TCGA-OPSCC expression dataset after variance filtering, and genes not represented in the final TCGA-OPSCC expression matrix. Hallmark gene sets were obtained from the Molecular Signatures Database (MSigDB). Gene sets were matched to the available expression features before ssGSEA. **Abbreviations:** ssGSEA, single-sample gene set enrichment analysis; OPSCC, oropharyngeal squamous cell carcinoma; MSigDB, Molecular Signatures Database; Tpex, precursor-exhausted T-cell-associated program.
(XLSX)

S2 Table Clinicopathological characteristics of the TCGA-OPSCC cohort according to HPV status. Available clinicopathological characteristics are summarized separately for HPV-negative (n = 26) and HPV-positive (n = 51) tumors. Values are presented as median [interquartile range] or n (%), as appropriate. Percentages for smoking status and clinical stage were calculated among cases with available data. Age was compared using the Wilcoxon rank-sum test, whereas sex/gender, smoking status, clinical stage, and anatomical subsite were compared using Fisher’s exact test. P values are unadjusted because these comparisons were performed for descriptive cohort characterization and were not included in the multiple-testing correction families used for transcriptional-program analyses.
(XLSX)

S3 Table HPV-associated transcriptional programs in the oropharynx-restricted TCGA-OPSCC cohort. **(A)** Unadjusted comparison of ssGSEA scores between HPV-positive (n = 51) and HPV-negative (n = 26) tumors in the oropharynx-restricted TCGA-OPSCC cohort. Effect sizes are reported as Cliff’s delta with bootstrap 95% confidence intervals. Positive values indicate higher scores in HPV-positive tumors. **(B)** Multivariable-adjusted associations between HPV status and transcriptional program scores among complete cases (n = 74; HPV-positive, n = 50; HPV-negative, n = 24). β represents the coefficient for HPV-positive status from linear models adjusted for age, sex/gender, smoking status, clinical stage, and anatomical subsite. Positive β values indicate higher scores in HPV-positive tumors. P values were adjusted across the eight prespecified transcriptional programs within each analysis using the Benjamini–Hochberg false discovery rate (BH-FDR) procedure. **(C)** Complementary multivariable-adjusted marker-gene analysis of the Stem-like T-cell-associated program among complete cases (n = 74; HPV-positive, n = 50; HPV-negative, n = 24). Eight prespecified marker genes (TCF7, IL7R, CCR7, LEF1, BACH2, SELL, SLAMF6, and CD28) were evaluated using linear models adjusted for age, sex/gender, smoking status, clinical stage, and anatomical subsite. β represents the coefficient for HPV-positive status. P values were adjusted across the eight marker genes using the Benjamini–Hochberg false discovery rate (BH-FDR) procedure. This analysis was considered complementary to, rather than an independent validation of, the predefined multigene Stem-like T-cell-associated program.
(XLSX)

S4 Table Patient-level analyses of transcriptional programs across GeoMx spatial compartments. **(A)** Within-patient comparisons of transcriptional programs between paired LFRs and TTRs in all six patients. Differences were evaluated using two-sided exact Wilcoxon signed-rank tests. The five immune-related programs were higher in LFRs than in TTRs in all six patients, whereas estrogen response early and late were higher in TTRs than in LFRs in all six patients (Benjamini–Hochberg false discovery rate [BH-FDR] = 0.036 for each). Bile acid metabolism showed no consistent compartment-specific direction. **(B)** Patient-level HPV-associated differences in ssGSEA scores within lymphoid follicular regions (LFRs) and tumor tissue regions (TTRs). Scores from six ROIs within each compartment were averaged for each patient, and HPV-positive (n = 4) and HPV-negative (n = 2) patients were compared using two-sided exact Wilcoxon rank-sum tests. Effect sizes are reported as Cliff’s delta, with positive values indicating higher scores in HPV-positive patients. None of the eight prespecified transcriptional programs showed a statistically significant HPV-associated difference within either compartment after BH-FDR correction. **(C)** HPV-by-compartment interaction analyses testing whether within-patient LFR-versus-TTR differences varied according to HPV status. Interaction-effect estimates and 95% confidence intervals were obtained from linear models, whereas statistical inference was based on exact patient-label permutation tests across all possible allocations of four HPV-positive and two HPV-negative patients. No interaction remained statistically significant after BH-FDR correction; estrogen response early showed the strongest suggestive interaction (exact permutation P = 0.067; BH-FDR = 0.533).
(XLSX)

S5 Table Reference-based cell-type deconvolution analyses of GeoMx lymphoid follicular regions. **(A)** Reference-based estimates of broad immune-cell composition within lymphoid follicular regions (LFRs) from six patients analyzed by GeoMx DSP. Cell-type abundances were estimated using SpatialDecon with the ImmuneTumor_safeTME reference and summarized at the patient level by averaging across six LFR ROIs per patient. HPV-positive (n = 4) and HPV-negative (n = 2) patients were compared using two-sided exact Wilcoxon rank-sum tests, with Benjamini–Hochberg false discovery rate (BH-FDR) correction across four prespecified broad cell-type summaries. No HPV-associated difference remained statistically significant after FDR correction. **(B)** Patient-level associations between the Stem-like ssGSEA score and deconvolution-derived estimates of B cells, CD4 T cells, CD8 T cells, and total T cells within LFRs. Associations were evaluated using Spearman rank correlations (n = 6). The Stem-like score was not positively associated with estimated CD8 T-cell proportion (ρ = −0.371, P = 0.468), whereas a positive association with estimated total T-cell proportion was observed (ρ = 0.829, P = 0.042). Given the small sample size, these correlation analyses were considered exploratory. **(C)** Exploratory patient-level linear models evaluating the HPV-associated direction of the Stem-like transcriptional program after adjustment for either estimated CD8 T-cell or total T-cell abundance. The HPV-associated direction was retained in both models, but confidence intervals were wide and the adjusted HPV coefficients were not statistically significant. These analyses do not establish composition-independent enrichment or a specific cellular origin of the Stem-like transcriptional program.
(XLSX)

S6 Table HPV-associated transcriptional patterns in tumor-free surrounding palatine tonsillar tissue. Patient-level ssGSEA scores for eight prespecified transcriptional programs were compared between HPV-positive (n = 23) and HPV-negative (n = 4) cases in tumor-free surrounding palatine tonsillar tissue (PTT). Effect sizes are reported as Cliff’s delta, with positive values indicating higher scores in HPV-positive cases and negative values indicating higher scores in HPV-negative cases. Group comparisons were performed using two-sided exact Wilcoxon rank-sum tests, with Benjamini–Hochberg false discovery rate (BH-FDR) correction across the eight programs. None of the evaluated transcriptional programs remained statistically significant after FDR correction; therefore, these analyses were considered exploratory.
(XLSX)

S7 Table Exploratory overall survival analyses of transcriptional programs in the TCGA-OPSCC cohort. **(A)** Univariable Cox proportional hazards analyses of overall survival in the TCGA-OPSCC cohort (n = 77; 21 deaths). Hazard ratios (HRs) are reported per 1-standard-deviation increase in each ssGSEA score. P values were adjusted across the eight prespecified transcriptional programs using the Benjamini–Hochberg false discovery rate (BH-FDR) procedure. Stem-like, Tpex, B-cell activity, and T-cell activity were significantly associated with improved overall survival after FDR correction, whereas the Tex-like program showed a nominal association that did not remain significant after correction. **(B)** Cox proportional hazards analyses including each standardized transcriptional program and HPV status. After accounting for HPV status, none of the eight transcriptional programs remained significantly associated with overall survival after BH-FDR correction. Schoenfeld residual-based tests showed evidence of non-proportional hazards for HPV status, motivating the HPV-stratified sensitivity analysis. **(C)** HPV-stratified Cox sensitivity analyses, allowing the baseline hazard to differ according to HPV status. None of the eight transcriptional programs was significantly associated with overall survival after BH-FDR correction. These analyses were exploratory and did not provide evidence that the evaluated transcriptional programs were associated with overall survival beyond the prognostic information captured by HPV status.
(XLSX)
